# Clinical Efficacy and Safety of Malarone^®^, Azithromycin and Artesunate Combination for Treatment of *Babesia gibsoni* in Naturally Infected Dogs

**DOI:** 10.3390/ani12060708

**Published:** 2022-03-11

**Authors:** Martina Karasová, Csilla Tóthová, Bronislava Víchová, Lucia Blaňarová, Terézia Kisková, Simona Grelová, Radka Staroňová, Alena Micháľová, Martin Kožár, Oskar Nagy, Mária Fialkovičová

**Affiliations:** 1 Small Animal Clinic, University of Veterinary Medicine and Pharmacy, 04001 Košice, Slovakia; simona.grelova@student.uvlf.sk (S.G.); radka.staronova@uvlf.sk (R.S.); ale.michalova@gmail.com (A.M.); martin.kozar@uvlf.sk (M.K.); maria.fialkovicova@uvlf.sk (M.F.); 2 Clinic of Ruminants, University of Veterinary Medicine and Pharmacy, 04001 Košice, Slovakia; csilla.tothova@uvlf.sk (C.T.); oskar.nagy@uvlf.sk (O.N.); 3 Institute of Parasitology, Slovac Academy of Sciences, 04001 Košice, Slovakia; vichova@saske.sk (B.V.); blanarova@saske.sk (L.B.); 4 Faculty of Science, University of Pavol Jozef Šafárik, 04180 Košice, Slovakia; terezia.kiskova@upjs.sk

**Keywords:** artesunate, atovaquone, azithromycin, *Babesia gibsoni*, babesiosis, Malarone^®^, treatment

## Abstract

**Simple Summary:**

*Babesia gibsoni* is an intracellular parasite of red blood cells that may cause anemia in dogs. Many drugs have been used in management of canine babesiosis such as monotherapy or combined treatment, but complete elimination of parasitemia was not proven, frequent relapses were observed and adverse effects occurred during treatment. This report examines the effectiveness and safety of Malarone^®^, azithromycin (AZM) and artesunate (ART) combination for the treatment of babesiosis in dogs naturally infected with *Babesia gibsoni*. The treatment improved hematology and biochemical parameters to the reference range. No clinically apparent adverse effects were reported during treatment and monitoring and no relapses of parasitemia were detected during monitoring until day 720 after treatment. Results of the study indicate that the combined treatment leads to successful elimination of parasitemia in chronically infected dogs with *B. gibsoni*.

**Abstract:**

*Babesia gibsoni* is a tick-borne protozoal blood parasite that may cause hemolytic anemia, thrombocytopenia, lethargy, and/or splenomegaly in dogs. Many drugs have been used in management of canine babesiosis such as monotherapy or combined treatment, including diminazene aceturate, imidocarb dipropionate, atovaquone, and antibiotics. This report examines the effectiveness and safety of Malarone^®^, azithromycin (AZM) and artesunate (ART) combination for the treatment of babesiosis in dogs naturally infected with *Babesia gibsoni*. Twelve American Pit Bull Terriers were included in the experiment. Examined dogs underwent clinical and laboratory analysis including hematology and biochemistry profile and serum protein electrophoresis. After diagnosis, the dogs received combined therapy with Malarone^®^ (13.5 mg/kg PO q24 h), azithromycin (10 mg/kg PO q24 h) and artesunate (12.5 mg/kg PO q24 h) for 10 days. The combined treatment improved hematology and biochemical parameters to the reference range gradually during the first 14 days already, resulting in the stable values until day 56 after treatment. No clinically apparent adverse effects were reported during treatment and monitoring. No relapses of parasitemia were detected in control days 180, 360, 540 and 720 in all dogs. Results of the study indicate that the combined treatment leads to successful elimination of parasitemia in chronically infected dogs with *B. gibsoni*.

## 1. Introduction

*Babesia gibsoni* is a vector-borne hemoprotozoan parasite of the genus *Babesia* which is distributed in areas of Asia, Africa, Middle East, Brazil, North America, Australia and Southern Europe [1]. In Slovakia, the presence of *B. gibsoni* was confirmed in 2016 [2]. This intraerythrocyte parasite induces hemolytic illness. Infection with *B. gibsoni* occurs following the bite of an ixodid tick from the genus *Haemaphysalis* and *Rhipicephalus* [3]. Non-vector transmissions have also been reported, including transplacental transmission, transmission by blood transfusion of contaminated blood or through infected dog bites. Infection with *B. gibsoni* is widespread among dog breeds commonly used for fighting such as American Pit Bull Terriers, American Staffordshire Terriers and Tosa dogs [4,5,6]. Transmission of babesial sporozoites into bloodstream of the canine host results in intra-erythrocytic multiplication and subsequent erythrocyte lysis, producing more parasites to infect intact erythrocytes [3]. A wide variety of pathological abnormalities including hemoglobinuria, hypoglycemia, azotemia, and elevation of liver enzymes levels are related to the extent of parasite replication in the host’s red blood cells with subsequent lysis [7]. Babesiosis caused by *B. gibsoni* is mostly chronic and asymptomatic. Natural or experimental *B. gibsoni* infections cause regenerative hemolytic anemia, thrombocytopenia, pyrexia, lethargy, icterus and splenomegaly [8]. The clinical presentation can vary from subclinical to severe disease resulting in multiple organ failure and death [3]. The chronic form is manifested by intermittent fever, lethargy, weight loss that can persist in the body for years [9]. Hematological changes are mostly represented by regenerative hemolytic anemia and thrombocytopenia [10,11]. Significant reductions in erythrocyte count and hemoglobin concentration are due to mechanical damage to erythrocytes during parasite migration out of the erythrocyte, intravascular hemolysis, and immune-mediated or non-immune-mediated destruction of erythrocytes [12,13]. Another confirmed mechanism that exacerbates anemia in *B. gibsoni* infected dogs is the erythrophagocytic capacity of peripheral blood and bone marrow macrophages [14]. The mechanism of the thrombocytopenia development is not yet fully understood. One possibility is platelet sequestration in the spleen or immune-mediated platelet destruction and the development of disseminated intravascular coagulopathy [12,13].

Many drugs have been used in management of canine babesiosis as a monotherapy or combined treatment, including diminazene aceturate, imidocarb dipropionate, atovaquone, and antibiotics, such as azithromycin (AZM), clindamycin, doxycycline, and metronidazole. Synergic or addictive effect of some of these drug combinations was evaluated, but complete elimination of parasitemia was not proven, frequent relapses were observed and adverse effects occurred during treatment [15,16,17,18,19,20,21].

The currently recommended treatment for *B. gibsoni* infection is administration of azithromycin (AZM) 10 mg/kg PO q24 h, in combination with atovaquone (ATV) 13.3 mg/kg PO q8 h for 10 days. The atovaquone–azithromycin drug combination seems to be an effective treatment for dogs that are chronically infected with *B. gibsoni* and results in either elimination of infection or the suppression of parasitemia below the limit of detection [22,23]. Although these therapeutic agents are beneficial and seem to partially provide a permanent cure, the treatment failed for some of the dogs and relapses occurred too [17]. Simultaneous use of atovaquone and azithromycin has an additive or synergic effect, whereas the use of atovaquone alone leads to recurrence of clinical signs of babesiosis. Nowadays, the combination of atovaquone (+/− proguanil hydrochloride) and azithromycin is considered to be the most effective for the treatment of *B. gibsoni* infection in dogs, although reduction of parasitemia below the level that can be detected by PCR is often insufficient. Despite the good effectiveness of this therapy, frequent recurrence of the disease is observed. Atovaquone is used in the treatment of *B. gibsoni*, *B. conradae* and *B. vulpes* infection in doses of 13.3 (or 13.5) mg/kg PO q8 h with a fatty meal, in combination with azithromycin in the dose of 10 mg/kg PO q24 h, both for 10 days [15,16,22,23,24,25,26].

Some studies have evaluated the effect of traditional Indonesian and Chinese plant extracts against *B. gibsoni* and the results of these studies are promising [17,27]. Artemisinin is extracted from *Artemisia annua* (*qinghao*), which is a Chinese annual herb. Artemisinin compounds, artesunate (ART), artemether and arteether are used as antimalarial drugs worldwide. Aside from its antimalarial and antischistosomal activity, it is also characterized by antimicrobial and antivirotic activity. Its antiparasitic effect includes a wide spectrum of protozoan parasites such as *Leishmania* spp., *Trypanosoma* spp., *Toxoplasma gondii*, *Neospora caninum*, *Eimeria tenella*, *Acanthamoeba castellanii*, *Naegleria fowleri*, *Cryptosporidium parvum*, *Giardia lamblia* and *Babesia* spp. [28]. In vitro growth inhibition of *B. gibsoni* with artesunate was evaluated to be more effective than some other drugs routinely used for babesiosis treatment. However, it is recommended to use ART in combination with other antiprotozoal drugs [17,29].

In the present study, we evaluated the efficacy of Malarone^®^ (atovaquone with proguanil hydrochloride), azithromycin and artesunate combination in chronic subclinical babesiosis of naturally infected dogs. At the same time, we assessed the presence of treatment side effects and relapse occurrence during the 24-month period after the treatment.

## 2. Materials and Methods

### 2.1. Dogs

Twelve dogs naturally infected with *B. gibsoni* were examined at the University Veterinary Hospital; University of Veterinary Medicine and Pharmacy in Košice, Slovakia. *B. gibsoni*-positive dogs were found through a vector-borne disease survey of 65 American Pit Bull Terriers belonging to sport kennels in Slovakia. Dogs were tested for a panel of infectious diseases using PCR assay and serology. Pregnant or lactating positive bitches and dogs with any vector-borne concomitant infection including *B. canis*, *Erlichia canis*, *Hepatozoon canis*, *Anaplasma* spp., *Leptospira* spp., *Leishmania infantum*, *Dirofilaria* spp., *Borrelia burgdorferi* or *Mycoplasma* spp. were excluded. The main criterion for inclusion of the samples into the study was *B. gibsoni* polymerase chain reaction (PCR) positivity. *B. gibsoni*-positive dogs had not received any treatment with antibiotics, antifungals, corticosteroids and/or a specific anti-*Babesia* agent within 180 days prior to PCR confirmation of *B. gibsoni*.

The dogs included in the study were American Pit Bull Terriers, there were 7 males and 5 females. The dog age ranged from 2 to 5 years old (2.1 ± 0.95 years) and their body weights ranged from 15.7 to 24.5 kg (20.35 ± 2.96 kg). Examined dogs underwent clinical and laboratory examination including hematology and biochemistry profile and serum protein electrophoresis. After diagnosis and clinical examination, the dogs received combination therapy with Malarone^®^, azithromycin and artesunate, and alterations in clinical parameters were evaluated. Hematological and biochemical analysis was performed on day 0 (pretreatment), 14, 28, 42, 56 and 720 along with PCR analysis at 0 (pretreatment), 14, 28, 42, 56, 180, 360, 540 and 720 days.

### 2.2. Evaluation of Clinical Parameters

All tested dogs underwent a thorough physical examination, blood counts and biochemical profiling along with parasite detection before (day 0) and after treatment (day 14, 28, 42, 56 and 720). Blood sampling was performed directly in the field of kennels by cephalic venipuncture, 1 mL of collected blood placed in EDTA tube for complete blood count and 1 mL for PCR and sequencing. The remaining 5 mL of blood was placed into tubes without anticoagulant for biochemical profiles and serum protein electrophoresis.

Complete hematological analysis was done using the ProCyte Dx automated hematology analyzer (IDEXX Laboratories, Westbrook, ME, USA). Biochemical parameters were analyzed with Cobas c 111 analyzer (Roche, Switzerland) and symmetric dimethylarginine (SDMA) with Catalyst One (IDEXX Laboratories, Westbrook, ME, USA).

The biuret method was applied to measure the total protein concentrations using commercially available diagnostic kits (Randox, Crumlin, UK) and the automated chemistry analyzer Alizé (Lisabio, Poully en Auxois, France). The separation and distribution of serum protein fractions were performed by zone electrophoresis on agarose gel using an automated electrophoresis system Hydrasys with commercial diagnostic kits Hydragel 7 Proteine (Sebia Corporate, Lisses, France) [30]. The protein fractions were quantified (g/L) from the total protein concentrations according to the optical density and percentage distribution of individual fractions on the electrophoretogram. Albumin/globulin (A/G) ratios were calculated as well.

### 2.3. Treatment Protocol and Monitoring

Dogs were treated with Malarone^®^ (13.5 mg/kg PO q24 h), azithromycin (10 mg/kg PO q24 h) and artesunate (12.5 mg/kg PO q24 h) for 10 days. Malarone^®^ (GlaxoSmithKline, SVK, Bratislava, Slovakia) is a medication containing 250 mg of atovaquone and 100 mg of proguanil hydrochloride (PG). The dosages and duration of the therapy with Malarone^®^ and azithromycin were based on published studies and further modified (ATV 17–25 mg/kg with PG 7–10 mg/kg q12 h, for 10 days [16]; ATV 13.3 mg/kg PO q8 h with a fatty meal and AZM 10 mg/kg PO q24 h, for 10 days [22,23]. Artesunate powder was compounded into gelatin capsules size 00. No other supportive or symptomatic treatment was used.

Response to the treatment was monitored by repeated PCR testing for *B. gibsoni* at 14, 28, 42 and 56 days. Treatment was considered to be successful if two PCR tests 14 days apart were negative. The day of negative PCR is specified as a day when the dog become *B. gibsoni* negative (day 14, 28, or 42, individually). Relapse of babesiosis was defined as the reappearance of *B. gibsoni* and further PCR sequencing was performed at 180, 360, 540 and 720 days. Hematological, biochemistry analysis, and serum protein electrophoresis were accomplished with PCR testing on day 720.

### 2.4. Molecular Analysis

The PCR diagnosis was based on initial amplification of a portion of 18S rRNA gene of *Babesia* spp. and subsequent amplification of the 18S rRNA of *B. gibsoni* in genomic DNA extracted from EDTA whole blood samples (Institute of Parasitology, Slovak Academy of Sciences). The PCR method used in the present study has enough sensitivity for detecting the DNA of *B. gibsoni* in the peripheral blood. Therefore, the negative result of the PCR shows the deletion of *B. gibsoni* [27,31].

Genomic DNA was extracted from 200 µL of EDTA-blood samples, using a commercial DNA extraction kit (GeneJET PCR Purification Kit, ThermoFisher Scientific, Vilnius, Lithuania). For the molecular detection of *Babesia* spp., PCR amplification of an approximately 450 bp long fragment of 18S rRNA gene, spanned by a reverse BJ1 (5′GTCTTGTAATTGGAATGATGG3′) and forward BN2(5′TAGTTTATGGTTAGGACTACG3′) primer was performed according to Casati et al. [32]. Only positive samples were further screened for the presence of *B. gibsoni* by PCR using the primers Gib599F (5′CTCGGCTACTTGCCTTGTC3′) and Gib1270R (5′GCCGAAACTGAAATAACGGC3′), which targeted a 671-bp long fragment of the 18S rRNA gene [33]. In each PCR reaction, sequenced DNA from *Babesia*-positive dog was used as positive control and nuclease-free water was added to the template in the negative control. The PCR products were visualized by electrophoresis on 1.5% agarose gels stained with GoodView Nucleic Acid Stain (Beijing SBS Genetech, Beijing, China). All positive PCR products were purified using a purification kit (Qiagen, Hilden, Germany) and sequenced. Nucleotide sequences were manually edited in MEGA X [34] and further compared with GenBank entries by BLAST [35].

### 2.5. Statistical Analysis

For statistical analyses, a GraphPad Prism 8.0 (GraphPad, San Diego, CA, USA) software package was used. A Student’s paired *t*-test for comparing the day 0 (pre-treatment day) with the day of negative PCR test (ranged between 14–48th day) and the day 56 was used. The values are expressed as mean ± SD. Statistical significance level was set to *p* < 0.05.

## 3. Results

### 3.1. Clinical Efficacy

The most prevalent clinical signs observed at initial presentation were pale mucous membranes (10/12; 83.3%), training weariness (9/12; 75%) and mild fever ≥ 39.5 °C (4/12; 33.3%). Other clinical signs were not reported (including anorexia, weight loss, jaundice, dark urine, diarrhea, melena, constipation, vomiting, polyuria/polydipsia, epistaxis, enlarged lymph nodes or petechiae). On day 56 no clinical signs including pale mucous membranes, training weariness and fever were observed. No clinically apparent adverse effects were reported during treatment and monitoring.

### 3.2. Clinicopathological Abnormalities

The laboratory parameters of all 12 dogs are summarized in Table 1. The laboratory analysis revealed anemia in 8/12 dogs (66%) before treatment, three of them were regenerative (3/8; 37%) and five nonregenerative (5/8; 62%). There were five cases of mild anemia and three cases of severe anemia with hematocrit ˂20% (HCT 12.7%, 16.5% and 18.7%, respectively), where the first mentioned case was of nonregenerative anemia. Red blood cells count, hematocrit and hemoglobin concentration increased significantly (*p* < 0.05) on the day of negative PCR (individual day for each dog). Thrombocytopenia was present in all cases of anemia (8/12; 66%). Platelet values increased significantly on the day of negative PCR test and day 56 after treatment (*p* < 0.01 and *p* < 0.05, respectively). White blood cells were not significant altered, therefore are not included in the table. Although leukocytosis (31.42 × 109/L), neutrophilia with left shift (21.09 × 109/L), lymphocytosis (6.16 × 109/L) and monocytosis (3.95 × 109/L) were present in the case with severe nonregenerative anemia (HCT 12.7%), the leucocyte count was within reference range on day 28.

Biochemistry analysis revealed a mild increase of ALT (in the range of 1.27–2.99 µkat/L) in 4/12 dogs (33%) and ALP in one dog (8%, 1.68 µkat/L). These values decreased gradually within the reference range in all cases, up to day 56 after treatment. The dog with most severe anemia had ALT 4.81 µkat/L and ALP 6.54 µkat/L before treatment. Although ALP initially had a tendency to decline, it reached a value of 12.82 µkat/L on day 56, but ALP decreased to the reference range. However, this dog was found to have ALT and ALP within the reference range on day 720. Bilirubinemia appeared in 3/12 dogs (25%) with severe anemia (21.7, 5.2 and 10.0 µmol/L, respectively) and were within the reference range during following sampling on day 14. The elevated value of total bilirubin was significantly lowered on the day of negative PCR (*p* < 0.01). Amylase was mildly increased in 6/12 dogs (50%), but was within reference range on day 14. Renal function parameters, SDMA, creatinine and urea, were not altered before treatment and remained that way throughout the monitoring.

### 3.3. Electrophoresis of Serum Proteins

**Figure 1:**
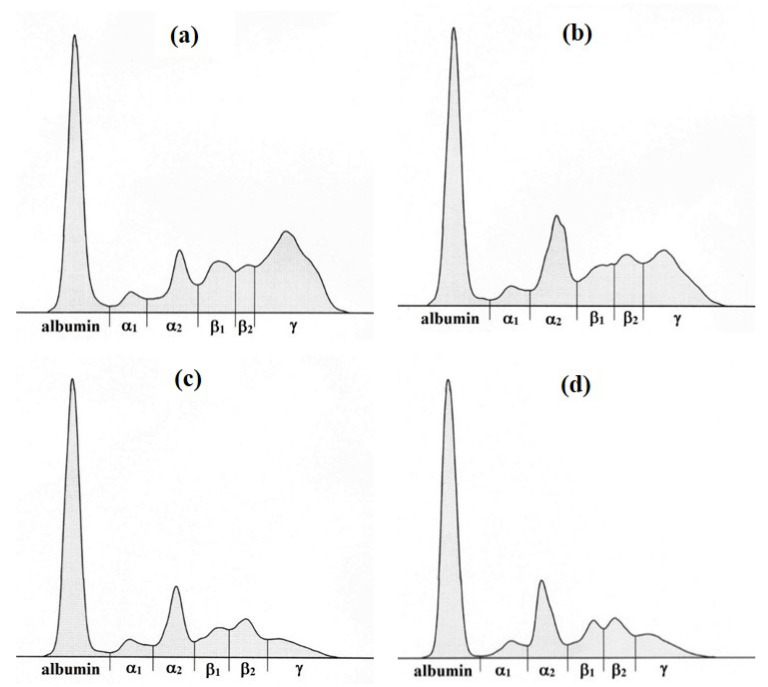
The representative electrophoretograms before (**a**) and following the treatment of *Babesia gibsoni* infection with Malarone^®^, azithromycin and artesunate combination ((**b**)—negative PCR test, (**c**)—day 56, (**d**)—day 720) of dog showing the serum protein fractions—albumin, α1-, α2-, β1-, β2- and γ-globulins.

Serum protein electrophoresis identified in dogs the following six protein fractions of different mobility: albumin, α1-, α2-, β1-, β2- and γ-globulins (Figure 1). As presented in Table 1, the highest mean total serum protein concentrations were recorded in dogs before treatment and on day 720. On the day when they produced negative PCR test for *B. gibsoni* and on day 56, the concentrations were significantly lower compared to those obtained before treatment (*p* < 0.001 and *p* < 0.05, respectively). The analyses of the concentrations of albumin showed significantly higher mean value on the day being PCR negative for *B. gibsoni* than that before treatment (*p* < 0.01), while the highest mean value was obtained on day 720 of treatment process.

The analyses of the concentrations of albumin showed significantly higher mean value on the day being PCR negative for B. gibsoni than that before treatment (*p* < 0.01), while the highest mean value was obtained on day 720 of treatment process. In the concentrations of α1-globulins, no marked differences were observed from day 0 till day 56 after treatment. Non-significantly higher mean value compared to day 0 was recorded in dogs on day 720 after treatment. Similarly, the concentrations of α2-globulins showed no marked differences between the day 0 and the evaluated periods of treatment process. In the concentrations of β1- and β2-globulins, non-significantly lower values compared to day 0 were found on the day when the dogs produced a PCR negative test for *B. gibsoni* and on day 56 after treatment. The values obtained on day 720 after treatment were comparable to those recorded before treatment. In the concentrations of γ-globulins, a trend of gradually decreasing values was observed during the treatment period, with the highest mean value before treatment and the lowest on day 720 after treatment. The A/G ratio in dogs before treatment was relatively low. Significantly higher mean values were recorded on day with PCR negativity and on day 56 after treatment (*p* < 0.001).

### 3.4. PCR Assay

The effect of treatment was monitored by PCR and results are summarized in Table 2. Initially, before the start of therapy, the presence of *B. gibsoni* was confirmed by PCR in 12 tested dogs. *B. gibsoni* DNA remained detectable by PCR in 7/12 dogs (58%) on day 14 and in 4/12 dogs (33%) on day 28 post-treatment, respectively. Table 2 shows that PCR assay realized on day 42 and 56 proved *B. gibsoni* negativity in all 12 samples. No relapses of parasitemia in those dogs were detected in control days 180, 360, 540 and 720, respectively.

*B. gibsoni*-positive amplicons obtained at the beginning of the screening were further sequenced. Altogether, 12 nucleotide sequences were manually edited, checked for any misreading and compared with GenBank entries by BLAST.

All nucleotide sequences of 18S rRNA gene fragment of *B. gibsoni* were submitted to GenBank database and are available under following Accession numbers: OM442901-08 and OM911924-27.

These nucleotide sequences were 100% identical with each other and showed 100% nucleotide sequence similarity (100% QC) with at least 100 different *B. gibsoni* isolates from dogs deposited in GenBank database, mainly from India, for example, Wayanad isolate 6 (MN134515), Trivandrum isolate 1 (MN134509), Kozhikode 20 (OK626641), Wd25 strain from Malaysia (MN068983), Chinese HZBg42 isolate (MN928828) [36], and/or Asian genotype Okinawa-2 from Japan (AB478328). Additionally, isolates from this study were identical within the overlapping region of 18S rRNA gene also with those, originally genotyped in Slovakia, KP737862-63 [2].

## 4. Discussion

In Slovakia, atovaquone is available in the combination with proguanil hydrochloride as Malarone^®^ and is commonly used in the prevention and treatment of malaria. The use of proguanil hydrochloride is accompanied with the occurrence of side effects in dogs such as gastrointestinal problems, and therefore the use of atovaquone as a separate drug (Mepron^®^, Atovaquone^®^) is recommended. Due to the high price of atovaquone and because it is not available in Slovakia, Malarone^®^ was used in present study. Given the side effects of Malarone^®^ and the authors’ previous clinical experience, lower daily dose, 13.5 mg/kg PO q24 h, was used. Azithromycin can cause gastrointestinal side effects too, such as diarrhea, vomiting, indigestion, stomach cramps and nausea [37]. In the present study, the dogs did not experience any digestive problems, including anorexia, vomiting and diarrhea, during treatment with Malarone^®^, azithromycin and artesunate combination, and the therapy was very well tolerated.

Artesunate is an ester derivative of artemisinin characterized by a good water solubility and a rapid onset of action [38]. Although there are no significant side effects of artemisinin and its derivatives observed in humans, the side effects in animals have been described in many studies. Artesunate therapy is characterized by having fewer side effects than any other artemisinin derivates, e.g., artemether and arteether. In a study of monkeys that were given a dose of 40 mg/kg artesunate PO for 14 days, only a reduced reticulocyte count was observed [39], similarly to our study, where the reticulocyte count was lower on the day of PCR negativity and the day 56, versus pretreatment day 0. The side effects of artesunate were also compared in another study in dogs, where low doses of artesunate (6 mg/kg) were given for a long period. Artesunate caused a decrease in erythrocyte count, extramedullary hematopoiesis in the liver and inhibition of erythropoiesis in the bone marrow; no neurotoxicity was present as with the use of artemether [40]. Although artesunate may reduce the number of RBCs, all dogs in the present study had a significant increase in RBCs on the day of negative PCR and remained in the reference range throughout the follow-ups. Even high doses of artesunate (50, 400, and 150 mg/kg) given orally to dogs for a short period of time only caused a reduction in gastric juice production and a decrease in gastric acidity, with no other observed cardiovascular, neurological, or respiratory changes [41]. Artesunate is therefore considered a very safe drug in terms of the development of side effects. The effect of artesunate on the elimination of *B. gibsoni* has been confirmed in vitro, but it is also recommended to increase its babesicidal effect in combination with other drugs [29]. In the present study, a dose of artesunate 12.5 mg/kg PO q24 h for 10 days was used, considered as a potentially safe dose, as no significant erythropoiesis-related changes occurred during monitoring.

The clinical signs of *B. gibsoni* infection are variable, depending not only on the level of parasitemia, but especially on the immune response of the infected dog [42]. The chronic form is manifested by intermittent fever, lethargy, weight loss and that can persist in the body for years [9]. Clinical signs observed at the initial presentation were pale mucous membranes, training weariness and fever, although owners previously did not realize that dogs had any pathological alterations, not even in dogs with severe anemia. In the present study, anemia and thrombocytopenia occurred in only 66% of dogs, but in other dogs the red blood cell count, hematocrit and hemoglobin concentration values were at the lower limit of the reference range. Although most of them were non-regenerative, anemia resolved in all dogs and RBC, HCT and HGB values were within reference range on day 56. Complicated forms of babesiosis can present with azotemia, acute renal failure, hepatopathy, icterus, pancreatitis and even multiorgan failure [43,44,45,46,47], but in the present study, only mild alterations in ALT and AMS were observed, which improved soon after the therapy.

It was stated previously that the infection with *B. gibsoni* in dogs causes marked alterations in serum biochemistry parameters, including those related to the protein profile that were characterized by lower concentrations of albumin and higher proportion of γ-globulins with low A/G ratio [48,49]. This pattern was observable also in the present study in dogs before treatment, reflecting the overproduction of γ-globulins as part of the humoral immune response of the organism to overcome the severe chronic inflammation caused by *B. gibsoni*. The most of published studies were aimed to describe the changes in the distribution of protein fractions in the Babesia infected dogs in comparison to healthy animals or determine the pathophysiological and biochemical changes in the blood occurring following the experimental infection with these parasites [50,51]. The alterations in the serum protein electrophoretic pattern during the treatment process are less well-documented. In the present study, significantly lower total serum protein concentrations were found on days when the dogs were considered PCR negative and on day 56 of treatment with significantly higher A/G ratio compared to pre-treatment values. Further analyses showed that the albumin and γ-globulin fraction contributed markedly to changes in total protein concentrations. The lower concentrations of total serum proteins after treatment were related to markedly decreasing values of γ-globulins in response to treatment. As a consequence of antigenic stimulation by the Babesia parasites, the infected animals produce immunoglobulins in high amounts. Immunoglobulins are the main constituents of the γ-globulin fraction and the increased antibody production against *B. gibsoni* (mainly IgM and IgG) was responsible for very high concentrations of γ-globulins in dogs before treatment [52]. The lower concentrations of γ-globulins in the period following the treatment may be attributed to gradually declined antibody levels as a response to treatment. An opposite trend was observed in the concentrations of albumin, being lower at the pre-treatment sample collection probably as a consequence of protein-losing nephropathy with glomerular leakage of proteins caused by the damage of renal cells by inflammatory mediators in the affected dogs [53]. The higher albumin concentrations obtained following the treatment may reflect positive therapeutic response. In the concentrations of α- and β-globulins, no significant differences among the sample collection in the period before and following treatment were found. These fractions contain many important proteins from the group of acute phase proteins. Their concentrations in the blood serum are very low and it is difficult to identify them electrophoretically [54]. The changes in their concentrations are usually not observable on the electrophoretogram and other methods or immunoenzymatic assays for the determination of individual serum proteins are recommended to evaluate which proteins are responsible for small alterations in the α- and β-globulin zones in dogs following the treatment of *B. gibsoni* infections. Seeing that there are very scarce literature data regarding the changes in the serum protein electrophoretic profile following the treatment of canine babesiosis caused by *B. gibsoni* parasite, further investigations would be helpful to understand the metabolic and biochemical processes occurring in the infected animals during the therapeutic process.

In all cases treated with the combination therapy, clinical signs of babesiosis, anemia, the number of platelets and biochemistry alterations improved soon after initiation of treatment. Although there were three dogs with severe anemia among the patients, no dog received a blood transfusion, intravenous fluids or other symptomatic therapy. PCR showed that 42% of the dogs were PCR negative on day 14 and in the others parasitemia was eliminated by the day 42. Relapses did not occur in any patient during treatment monitoring (720 days), despite the fact that the dogs were under intense training load and participated in sport competitions. Thus, hematological and biochemical analysis of dogs showed that all parameters were within reference range on day 720.

Since atovaquone (Mepron^®^, Atovaquone^®^) is not accessible in many countries, Malarone^®^ is available worldwide as an antimalarial drug. The important aspect of using Malarone^®^ instead of atovaquone is its price, which is significantly lower, thus the effective treatment could also be more available for dogs in shelters confiscated for illegal dog fights [23,55]. The cost, accessibility and effectiveness of the Malarone^®^, azithromycin and artesunate treatment is advantageous for application in clinical cases.

## 5. Conclusions

This study suggested that a combination of Malarone^®^, azithromycin and artesunate is an effective treatment for dogs that are chronically infected with *B. gibsoni*. It is the only treatment for *B. gibsoni* infection that showed complete elimination of parasitemia below the limit of PCR detection with no relapses and adverse effects occurrence in a long period of monitoring.

## Figures and Tables

**Table 1:** Hematological and biochemical parameters in 12 dogs showing clinical remission for babesiosis following treatment with Malarone^®^, azithromycin and artesunate combination.

Parameter	Reference Range	Day 0		PCR Negative		Day 56		Day 720	
		OOR	Mean ± SD	OOR	Mean ± SD	OOR	Mean ± SD	OOR	Mean ± SD
RBC, 1012/L	5.65–8.87	8	4.76 ± 2.46 •	3	8.04 ± 0.94 *	0	6.69 ± 1.07	0	7.84 ± 0.33
HCT, %	37.3–61.7	8	33.25 ± 15.80 •	3	49.44 ± 4.41 *	0	41.68 ± 6.31	0	48.50 ± 2.58
HGB, g/dL	13.1–20.5	8	11.51 ± 5.41 •	3	16.51 ± 1.38 *	0	14.09 ± 2.34	0	17.28 ± 0.10
RDW, %	13.6–21.7	4	20.01 ± 2.76	3	21.23 ± 2.03	1	20.52 ± 1.91	0	18.18 ± 0.34
RET, K/µL	10.0–110.0	3	99.14 ± 73.02	4	50.39 ± 16.80	0	64.09 ± 50.71	0	80.28 ± 50.27
PLT, K/µL	148–484	8	151.50 ± 102.20	3	280.10 ± 100.70 **	0	318.30 ± 178.80 *	0	291.25 ± 31.48
ALT, µkat/L	˂0.949	5	1.39 ± 1.28 •	2	2.50 ± 4.38 •	1	1.00 ± 1.15	1	0.94 ± 0.14
ALP, µkat/L	˂1.24	3	1.25 ± 1.74	0	0.65 ± 0.29	ND	ND	0	0.50 ± 0.36
T BIL, µmol/L	˂3.1	3	5.29 ± 6.55 •	0	0.37 ± 0.64 **	0	0.80 ± 0.94 *	0	1.45 ± 1.22
AMS, µkat/L	˂7.21	6	7.34 ± 2.36 •	0	7.07 ± 2.74	0	6.33 ± 2.10	0	5.35 ± 0.94
SDMA, µg/dL	0–14	1	9.90 ± 2.42	ND	ND	0	7.42 ± 2.28	0	8.16 ± 2.11
CREA, µmol/L	46–88	0	49.80 ± 14.67	1	64.69 ± 15.97	0	61.48 ± 20.39	0	53.00 ± 4.10
BUN, mmol/L	3.97–8.05	0	3.92 ± 1.44	0	3.98 ± 0.79	0	4.16 ± 1.47	0	4.97 ± 1.16
TP, g/L	47–74	4	72.90 ± 6.29	1	64.23 ± 2.28 ***	2	66.59 ± 7.62 *	1	73.18 ± 3.45
ALB, g/L	26–41	6	27.03 ± 4.75	3	32.64 ± 3.14 **	0	30.18 ± 4.58	0	36.65 ± 4.57
Alpha 1, g/L		ND	2.77 ± 0.46	ND	2.59 ± 0.42	ND	2.61 ± 0.43	ND	3.23 ± 0.19
Alpha 2, g/L		ND	8.45 ± 1.60	ND	10.04 ± 2.01	ND	9.39 ± 1.83	ND	10.13 ± 0.99
Beta 1, g/L		ND	7.12 ± 1.70	ND	6.51 ± 2.31	ND	6.88 ± 4.34 *	ND	7.55 ± 1.05
Beta 2, g/L		ND	7.56 ± 1.86	ND	6.50 ± 1.60	ND	6.88 ± 1.20	ND	7.95 ± 0.97
Gamma, g/L		ND	17.75 ± 9.45	ND	13.61 ± 17.35 *	ND	10.00 ± 6.41 **	ND	7.65 ± 2.00
A/G ratio	0.6–1.1	9	0.47 ± 0.13 •	2	0.93 ± 0.21 ***	0	0.95 ± 0.32 *	0	1.02 ± 0.20

Data are expressed as mean ± SD. Significance versus pretreatment day 0: * *p* < 0.05; ** *p* <0.01; *** *p* < 0.001. Values out of reference range are marked as •. ND, not determined; Day PCR negative (day 14, 28 or 42, individually); OOR; number of dogs that had values out of the reference range; RBC, red blood cells; HCT, hematocrit; HGB, hemoglobin; RDW, red cell distribution width; RET, reticulocyte count; PLT, platelet counts; ALT, alanine amino transferase; ALP, alkaline phosphatase; T BIL, total bilirubin; AMS, amylase; SDMA, symmetric dimethylarginine; CREA, creatinine; BUN, blood urea nitrogen; TP, total protein; ALB, albumin; A/G; albumin/globulin ratio.

**Table 2:** Results of polymerase chain reaction (PCR) testing for *Babesia gibsoni* in 12 dogs following the treatment with Malarone^®^, azithromycin and artesunate combination, divided into groups according to anemia occurrence.

Day	PCR+ (%)								
	0	14	28	42	56	180	360	540	720
Severe anemia *n* = 3	3/3 (100)	1/3 (33)	1/3 (33)	0/3 (0)	0/3 (0)	0/3 (0)	0/3 (0)	0/3 (0)	0/3 (0)
Mild anemia *n* = 5	5/5 (100)	4/5 (80)	1/5 (20)	0/5 (0)	0/5 (0)	0/5 (0)	0/5 (0)	0/5 (0)	0/5 (0)
Without anemia *n* = 4	4/4 (100)	2/4 (50)	2/4 (50)	0/4 (0)	0/4 (0)	0/4 (0)	0/4 (0)	0/4 (0)	0/4 (0)
Total	12/12 (100)	7/12 (58)	4/12 (33)	0/12 (0)	0/12 (0)	0/12 (0)	0/12 (0)	0/12 (0)	0/12 (0)

## Data Availability

All data are provided in the article.
